# Comparison of 2005 and 2024 diagnostic criteria for early identification of pediatric sepsis and septic shock in PICU patients: a prospective cohort study

**DOI:** 10.3389/fped.2025.1748925

**Published:** 2026-02-03

**Authors:** Wei Li, Haiyan Ge, Jin Zhang, Ning Li, Xiuxiu Lu, Jing Chen, Dong Qu, Shuang Liu, Chuanhe Liu

**Affiliations:** 1 Department of Critical Care Medicine, Capital Center for Children’s Health, Capital Medical University, Capital Institute of Pediatrics, Beijing, China; 2 Department of Critical Care Medicine, Capital Institute of Pediatrics, Chinese Academy of Medical Sciences & Peking Union Medical College, Beijing, China; 3 Department of Allergy, Capital Center for Children’s Health, Capital Medical University, Capital Institute of Pediatrics, Beijing, China; 4 Department of Allergy, Capital Institute of Pediatrics, Chinese Academy of Medical Sciences & Peking Union Medical College, Beijing, China

**Keywords:** diagnostic criteria, early recognition, pediatric sepsis, phoenix sepsis score (PSS), septic shock

## Abstract

**Background:**

In 2024, new international consensus criteria for pediatric sepsis and septic shock (2024 criteria) were introduced, replacing the 2005 criteria. The 2024 criteria use the Phoenix Sepsis Score (PSS) to define sepsis (score ≥2) and septic shock (cardiovascular PSS ≥1) in children with suspected infection, moving away from the 2005 reliance on systemic inflammatory response syndrome (SIRS). This study compares the two criteria in terms of diagnostic consistency, disease severity, prognosis, and early identification.

**Methods:**

Pediatric patients with infection admitted to the PICU at the Capital Institute of Pediatrics from May 2023 to May 2025 were prospectively enrolled. Those diagnosed with sepsis within 0–6 h of admission were included. Data on demographics, infection sites, pathogens, laboratory markers (platelets, albumin, creatinine, lactate), organ dysfunction scores (PCIS), and clinical outcomes (mechanical ventilation, CRRT, MODS, DIC, mortality) were collected. Diagnostic agreement was assessed using Kappa statistics, and performance was compared using McNemar's test. The 2005 criteria served as the reference for calculating sensitivity, specificity, and predictive values of the 2024 criteria.

**Results:**

The 2024 criteria identified fewer sepsis (80 vs. 240) and septic shock (49 vs. 86) cases. Diagnostic agreement was poor (Kappa = 0.161, *P* < 0.001), with significant differences in severity markers (lactate, PCIS, MODS) and outcomes. The 2024 criteria better reflected sepsis severity but were associated with potential underdiagnosis of early septic shock. For septic shock, 20 cases met vasoactive criteria only, risking missed early diagnosis. Cardiovascular thresholds in the 2024 PSS may be overly strict, delaying recognition. No significant difference in predicted mortality was observed between criteria.

**Conclusions:**

The 2024 sepsis criteria improve specificity but may overlook early septic shock. The 2024 septic shock criteria are stricter, potentially delaying diagnosis and treatment. Prospective studies and AI-supported early warning models are needed for better early identification and outcomes.

## 1 Introduction

In early 2024, the 2024 International Consensus Criteria for Pediatric Sepsis and Septic Shock(hereafter referred to as the “2024 Diagnostic Criteria”) (1) were published, with the aim of replacing the 2005 version of the pediatric septic shock diagnostic criteria (hereafter referred to as the “2005 Diagnostic Criteria”) (2). A key distinction between the two versions is that the 2024 criteria no longer rely on infection-related systemic inflammatory response syndrome (SIRS) as the foundation. Instead, they focus on the pathophysiological core of “dysregulated host response to infection,” emphasizing the life-threatening organ dysfunction caused by this dysregulation. Specifically, the 2024 criteria define that a child with suspected infection can be confirmed to have sepsis if their Phoenix Sepsis Score (PSS) — an assessment tool covering dysfunction in the respiratory, cardiovascular, coagulation, and neurological systems — scores ≥2 points. Among these sepsis cases, those with a PSS cardiovascular subscore of ≥1 point are diagnosed with septic shock. In clinical practice, substantial discrepancies have been observed between the 2005 and 2024 criteria in the diagnosis of sepsis and septic shock. These differences can directly influence clinicians’ diagnostic decisions and subsequent therapeutic strategies, thereby affecting patient outcomes. Accordingly, this study compares the two diagnostic criteria with respect to the early identification, diagnosis, and prognostic evaluation of pediatric sepsis and septic shock, with the objective of informing evidence-based implementation of these diagnostic frameworks in clinical practice.

## 2 Methods

### 2.1 Study design and participants

We conducted a comparison study of pediatric patients (aged >28 days to ≤18 years) with suspected or confirmed infection admitted to the PICU of Capital Center for Children's Health, Capital Medical University in China between May 1, 2023, and July 31, 2024. Inclusion required sepsis diagnosis according to the 2005 international pediatric sepsis consensus (2) within 6 h of admission. Patients were excluded if they had known or suspected cardiac disease, preexisting hypertension, diabetes mellitus, or thyroid disorders, received vasoactive agents for indications other than shock, had incomplete clinical data, or if their guardians refused to provide informed consent for study participation. The study protocol was approved by the Ethics Committee of the Capital Institute of Pediatrics (Approval No.: SHERLL2023046). All procedures were conducted in strict accordance with the Declaration of Helsinki and the Ethical Review Measures for Biomedical Research Involving Humans (2016 Edition). For children under 7 years of age, with confirmation from their legally authorized representatives, researchers provided a verbal explanation of relevant study information, including the purpose, procedures, potential risks, and the rights of the child. Details of the disclosure and the guardian's acknowledgment were recorded in the medical records, which was deemed as completion of the informed consent process. For children aged 7 years and above diagnosed with sepsis or septic shock, due to the critical nature of their condition, they may experience impaired consciousness and are unable to provide independent informed consent. Therefore, this study waived the requirement for obtaining informed consent from children aged ≥7 years.

### 2.2 Data collection

Data were prospectively collected via the electronic medical record (EMR) system within 6 h of ICU admission. Data collection protocol included: (1) Baseline clinical characteristics: age, sex, vital signs, infection source, medical history, and comorbidities; (2) Physiological and laboratory parameters: (1) complete blood count, (2) hepatic/renal function tests, (3) coagulation profiles, (4) arterial blood gas analysis, (5) blood/urine/stool cultures, (6) pathogen detection assays and (7) pediatric critical illness score (PCIS), (8) pediatric risk of mortality IV score(PRISM IV), (9) pediatric index of mortality version 3(PIM-3); (3) Assessment of whether the patient developed disseminated intravascular coagulation (DIC) or multiple organ dysfunction syndrome (MODS), use of mechanical ventilation or continuous renal replacement therapy (CRRT), and clinical outcomes.

### 2.3 Diagnostic criteria

This study applied two sets of diagnostic criteria. The 2005 diagnostic criteria (2) defined sepsis as a systemic inflammatory response syndrome (SIRS) resulting from infection, and septic shock as sepsis accompanied by tissue hypoperfusion and cardiovascular dysfunction. The 2024 diagnostic criteria (1) defined sepsis as a Phoenix Sepsis Score (PSS)—assessing dysfunction across the respiratory, cardiovascular, coagulation, and neurological systems—in a child with suspected or confirmed infection that was ≥2 points, and septic shock as sepsis with a PSS cardiovascular subscore of ≥1 point.

### 2.4 Statistical analysis

Statistical analysis was performed using SPSS software, version 26.0. (1) Categorical variables are presented as number of cases (percentage) and compared between groups using the chi-square test. (2) Non-normally distributed continuous variables are expressed as median (interquartile range) and compared using the Mann–Whitney *U* test. (3) Agreement between the two diagnostic criteria was assessed using the Kappa statistic; diagnostic performance was evaluated using the McNemar test; and intergroup comparisons were conducted using the chi-square test. (4) Using the 2005 diagnostic criteria as the reference standard, the sensitivity, specificity, positive predictive value, and negative predictive value of the 2024 diagnostic criteria were calculated. A Pvalue of less than 0.05 was considered statistically significant.

## 3 Results

### 3.1 Patients characteristics

A total of pediatric patients with sepsis admitted to the Department of Critical Care Medicine at the Capital Institute of Pediatrics, affiliated with Capital Medical University, between May 2023 and May 2025 were included. Baseline patient information is presented in Table 1 (based on the 2005 diagnostic criteria) and Table 2 (based on the 2024 diagnostic criteria).

**Table 1:** Baseline characteristics nof enrolled cases according to the 2005 diagnostic criteria.

Clinical values		Sepsis (*n* = 240)	Non-shock Sepsis (*n* = 154)	Septic Shock (*n* = 86)	*χ*^2^/H	*P*-value
Age [median (Q1, Q3)], months	12 (3,58.25)	7 (3,23.25)	42.50 (4.75,90.25)	26.674	<0.001	
Sex, No. (%)					2.683	>0.05
	Male	131 (54.6%)	78 (50.6%)	53 (61.6%)		
	Female	109 (45.4%)	76 (49.4%)	33 (38.4%)		
Presence of underlying disease(s)		73 (30.4%)	27 (17.5%)	46 (53.5%)	33.708	<0.001
Infection Site	Bloodstream	15	3	12	60.800	<0.001
	Respiratory	125	99	26		
	Urinary	38	31	7		
	Central Nervous	15	9	6		
	Multiple system	21	6	15		
	Other Systems	26	6	20		
Pathogen	Bacteria	98	53	45	14.543^a^	0.004
	Virus	77	53	24		
	Fungus	3	1	2		
	Mycoplasma	23	21	2		
	Other	39	26	13		

Data conforming to a normal distribution are represented by mean ± standard deviation (SD), and data not conforming to a normal distribution are represented by median (Q1, Q3). *Statistically significant compared with the decompensated septic shock group. ^&^Statistically significant compared with the compensated septic shock group. ^#^No significant difference compared with the non-shock sepsis group. ^a^Fisher's exact test.

**Table 2:** Baseline characteristics of enrolled cases according to the 2024 diagnostic criteria.

Clinical values		Sepsis (*n* = 80)	Non-shock sepsis (*n* = 31)	Septic shock (*n* = 49)	*χ*^2^/H	*P*-value
Age [median (Q1, Q3)], months	60 (24.50,112.25)	31 (12,68)	76 (42.5,131)	−3.457	0.001	
Sex, no. (%)					1.064	>0.05
	Male	47	16 (51.6%)	31 (63.3%)		
	Female	33	15 (48.4%)	18 (36.7%)		
Presence of underlying disease(s)		45 (56.3%)	10 (32.3%)	35 (71.4%)	13.79	<0.001
Infection Site	Bloodstream	13	3	10	35.294^a^	<0.001
	Respiratory	27	21	6		
	Urinary	3	3	0		
	Central Nervous	4	0	4		
	Multiple system	14	3	11		
	Other Systems	19	1	18		
Pathogen	Bacteria	44	12	32	11.416^a^	0.013
	Virus	14	8	6		
	Fungus	2	0	2		
	Mycoplasma	7	6	1		
	Other	13	5	8		

Data conforming to a normal distribution are represented by mean ± standard deviation (SD), and data not conforming to a normal distribution are represented by median (Q1, Q3). *Statistically significant compared with the decompensated septic shock group. ^&^Statistically significant compared with the compensated septic shock group. ^#^No significant difference compared with the non-shock sepsis group. ^a^Fisher's exact test.

According to the 2024 diagnostic criteria, among the 49 pediatric patients diagnosed with septic shock, 20 required the addition of 1 point due to the use of vasoactive agents within the cardiovascular system component of the PSS in order to meet the diagnostic threshold for septic shock.

### 3.2 Analysis of diagnostic agreement between the two diagnostic criteria for sepsis and septic shock

The two diagnostic criteria showed poor agreement in the diagnosis of sepsis and septic shock (Kappa value = 0.161, *P* < 0.05). The detailed diagnostic results are shown in Table 3.

**Table 3:** The diagnostic results for sepsis and septic shock according to the two diagnostic criteria.

2024-criteria 2005-criteria	Non-sepsis	Non-shock sepsis	Septic shock	Total
Non-sepsis	0	0	0	0
Non-shock sepsis	133	21	0	154
Septic shock	27	10	49	86
Total	160	31	49	240

### 3.3 Analysis of diagnostic agreement between the two diagnostic criteria for septic shock

The two diagnostic criteria showed poor agreement in the diagnosis of septic shock (Kappa value = 0.630, *P* < 0.001), and the difference in diagnostic performance was statistically significant (McNemar test, *P* < 0.001). Using the 2005 diagnostic criteria as the reference standard, the 2024 diagnostic criteria for septic shock demonstrated a sensitivity of 57.0%, a specificity of 100%, a positive predictive value (PPV) of 100%, and a negative predictive value (NPV) of 80.6%. The detailed diagnostic results are shown in Table 4.

**Table 4:** The diagnostic results for septic shock according to the two diagnostic criteria.

2024-criteria 2005-criteria	Positive	Negative	Total
Positive	49	37	86
Negative	0	154	154
Total	49	191	240

### 3.4 Analysis of differences between the two diagnostic criteria in the severity and prognosis of septic shock

## 4 Discussion

Sepsis is a life-threatening organ dysfunction caused by a dysregulated host response to infection, whereas septic shock is a severe subtype of pediatric sepsis characterized by profound circulatory dysfunction and cellular metabolic abnormalities, and is associated with a higher risk of mortality (3). A global survey published in The Lancet in 2020 revealed that sepsis was responsible for the deaths of 2.9 million children under 5 years of age, 454,000 children aged 5–19 years, and adolescents worldwide (4). In China, a multicenter retrospective study involving 10 pediatric intensive care units (PICUs) and a prospective cross-sectional study involving 53 hospitals reported mortality rates of 23.3% and 18.3%, respectively, among children with septic shock (5, 6), indicating that septic shock continues to account for a significant proportion of pediatric deaths. The progression from sepsis to septic shock occurs over a very short period, and it can even happen within 24 h. Early identification and effective early intervention are of great significance for reducing mortality, and early identification and diagnosis are the key to early intervention. However, the findings of this study reveals a poor level of agreement between the 2005 and 2024 versions of the diagnostic criteria for pediatric sepsis and septic shock. Significant differences were observed in case inclusion scope, efficiency of severe condition identification, and capacity for early diagnosis. Specifically, the number of sepsis cases identified according to the two sets of criteria was 240 and 80, respectively, while the probability of progression from sepsis to septic shock was 35.8% under the 2005 criteria and 61.3% under the 2024 criteria. This discrepancy has a direct impact on the early diagnosis and treatment of pediatric sepsis and septic shock. Therefore, standardized diagnosis of sepsis and septic shock is of critical importance, particularly the establishment of diagnostic criteria tailored to the physiological characteristics of Chinese children, which directly influences treatment strategies and outcomes in this population in China.

With advances in the pathophysiology of sepsis, the definitions and diagnostic criteria for sepsis and septic shock in adults have been continuously updated and refined (3, 7, 8). However, the definition and diagnostic criteria for pediatric sepsis and septic shock have continued to follow the 2005 version, with no breakthrough progress, until the Society of Critical Care Medicine (SCCM) in the United States issued the new pediatric diagnostic criteria in 2024. The 2005 pediatric sepsis definition is based on the presence of infection plus systemic inflammatory response syndrome (SIRS), which has been widely used in clinical practice. However, the SIRS criteria are overly broad and lack specificity. On the one hand, they fail to effectively identify children at high risk of severe illness; on the other hand, they are prone to overdiagnosis and overtreatment, thereby limiting their utility in accurate prognostic assessment. In 2024, SCCM in the United States issued new diagnostic criteria for pediatric sepsis and septic shock (hereafter referred to as the “2024 diagnostic criteria”). These criteria were developed using in-hospital mortality as the primary outcome and employed a stacked regression modeling approach. For children with suspected infection, the PSS—which evaluates dysfunction across four organ systems (respiratory, cardiovascular, coagulation, and neurological)—is used to diagnose sepsis and septic shock (1).The PSS focuses on predicting mortality and emphasizes sepsis-associated organ dysfunction. Each of the four systems assessed by the PSS has clearly defined numerical scoring ranges, thereby reducing clinician subjectivity, improving the accuracy of organ function assessment, and enhancing diagnostic specificity. However, when scoring the cardiovascular system, the diagnosis of septic shock requires either a lactate level ≥5 mmol/L or the use of vasoactive agents. In clinical practice, some cases of early-stage septic shock may not meet these criteria, potentially resulting in missed diagnoses. Conversely, by the time a pediatric patient with sepsis has a cardiovascular PSS score of ≥1—which is required for the diagnosis of septic shock under the 2024 criteria—the condition may have already progressed significantly, possibly leading to MODS and further complicating treatment and management.

A further comparison of the specific differences between the two versions of diagnostic criteria in the diagnosis of sepsis and septic shock reveals that their core areas of discrepancy differ in emphasis. Studies show that, in the comparison of sepsis diagnosis, among the 154 pediatric sepsis patients without shock diagnosed by the 2005 criteria, only 21 would still meet the criteria for sepsis under the 2024 version. This is likely related to the overly broad SIRS-based diagnostic criteria in the 2005 version, which fails to effectively identify severe sepsis cases at risk of progressing to septic shock and is prone to resulting in overtreatment. In contrast, the 2024 version employs a multi-system quantitative assessment using the Pediatric Sequential Organ Failure Assessment (pSOFA) score, enabling more precise identification of high-risk sepsis patients and thereby reducing unnecessary medical interventions. In the comparison of septic shock diagnosis, among the 86 pediatric patients diagnosed with septic shock according to the 2005 criteria, only 49 would still qualify under the 2024 version. Notably, 20 of these 49 cases did not meet the 2024 septic shock diagnostic thresholds based on elevated lactate levels or decreased mean arterial pressure throughout the disease course; they could only be classified as septic shock after receiving vasoactive agents (scoring 1–2 points). However, considering the growth and developmental characteristics of children, effective circulating volume loss may reach as high as 35% even before hypotension occurs (i.e., prior to vasoactive agent administration). The onset of hypotension often indicates that circulatory failure has already progressed to an advanced stage (9). This suggests that the 2024 diagnostic criteria may be overly strict, potentially resulting in the underdiagnosis of septic shock in its early stages and thereby delaying appropriate treatment.

These findings highlight the clinical challenges posed by the differences between the two sets of criteria and underscore the importance of early recognition in pediatric septic shock. In response to such concerns, the Expert Consensus on the Management of Pediatric Septic Shock (2025) (10), developed by the Emergency Medicine Group of the Chinese Pediatric Society, the Pediatric Group of the Chinese Society of Emergency Medicine, and the Editorial Board of the Chinese Journal of Pediatrics, recommends close monitoring of tissue perfusion in children with suspected or confirmed infection. This includes assessing key clinical indicators such as consciousness, urine output, skin appearance, lactate levels, and capillary refill time. Our own retrospective review of cases within our department supports this recommendation. The 20 children who only met the 2024 septic shock criteria after receiving vasoactive agents had already exhibited clear signs of impaired tissue perfusion prior to the initiation of such therapy. However, their lactate levels and blood pressure remained below the thresholds required by the PSS, further emphasizing the critical need for early recognition beyond reliance on prognostic scoring alone. Further analysis of the cardiovascular scoring component of the PSS reveals several potential limitations that may hinder early diagnosis of septic shock. According to the 2024 PSS, a diagnosis of septic shock based on cardiovascular dysfunction requires either a MAP below specific age-related thresholds (range: under 1 month ≤30 mmHg; 1–11 months≤38 mmHg; aged 1–<2 years ≤43 mmHg; aged 2–<5 years ≤44 mmHg; aged 5–<12 years ≤48 mmHg; aged 12–<18 years ≤51 mmHg), the use of at least one vasoactive agent, or a lactate level ≥5 mmol/L. While these criteria aim to improve diagnostic specificity, they present significant challenges in clinical practice. First, the MAP thresholds set for each pediatric age group appear relatively low. For instance, the 50th percentile MAP for a 5-year-old child is approximately 62.5 mmHg, yet a value of ≤48 mmHg is required to assign a single point on the PSS. Second, the requirement of at least one vasoactive agent indicates that the patient is already beyond the early phase of shock, as the need for such support typically reflects inadequate fluid resuscitation and established hemodynamic instability. Third, a lactate level ≥5 mmol/L may not be present in all cases of septic shock, including some early or even later stages, and in many instances lactate levels may remain normal or only mildly elevated. Relying on this threshold may therefore delay diagnosis, particularly in cases where early intervention is most critical. Once lactate does reach ≥5 mmol/L, it often signals a more advanced and severe state of shock with significant organ and tissue hypoperfusion. Considering that the cardiovascular function component of the 2024 PSS scoring system is overly stringent, it may fail to enable early identification of septic shock. It is possible that, as the disease progresses, a child begins to receive vasoactive agents or exhibits progressively elevated lactate levels that eventually meet the diagnostic threshold; however, this approach runs counter to the principle of early recognition and prompt intervention in septic shock.

Statistical analysis of disease severity and prognostic indicators in pediatric patients under the two versions of diagnostic criteria further corroborates the aforementioned discrepancies (Tables 5, 6). Among children with sepsis, significant differences were observed between the two criteria in platelet count, albumin, creatinine, and lactate levels. Moreover, marked differences were also found in indicators reflecting disease severity and prognosis — including DIC, MODS, PCIS, PRISM IV score, PIM-3 score, mechanical ventilation use, and overall outcome. These findings suggest that the 2024 version of the diagnostic criteria more accurately reflects the severity of sepsis, while mitigating the problem of overtreatment associated with the 2005 criteria. In children with septic shock, the two versions differed only in platelet count and albumin levels, with no statistically significant differences in creatinine or lactate levels. Among the severity and prognostic indicators, differences were noted in MODS, PRISM IV score, and PIM-3 score; however, there were no differences in DIC, PCIS score, mechanical ventilation requirement, CRRT, or outcome. This indicates that although child with septic shock diagnosed by the different criteria may vary to some extent in disease severity, the 2024 version does not demonstrate differences in major clinical treatments (mechanical ventilation, CRRT) or final prognostic outcomes. Combined with our findings, this suggests that the 2024 criteria may be unfavorable for the early identification of pediatric septic shock.

**Table 5:** Comparison of disease severity and prognosis of sepsis between the two diagnostic criteria.

Clinical values	2005-criteria (*n* = 240)	2024-criteria (*n* = 80)	χ^2^/Z	*P*-value
Platelet count (×10^9^ /L)	303.0 (186.3,469.8)	110 (23.3,246.3)	−6.062	<0.001
Albumin (g/L)	37.4 (33.0,40.9)	32 (28.8,34.8)	−6.479	<0.001
ALT (U/L)	22.1 (16.2,38.4)	27.4 (16.3,58.8)	−1.716	>0.05
Creatinine (μmol/L)	23.5 (19.6,29.8)	27.9 (20.6,49.2)	−2.789	0.005
Lactate ≥2 mmol/L	90 (37.5%)	47 (58.6%)	11.066	0.001
Lactate ≥5 mmol/L	18 (7.5%)	17 (21.3%)	11.645	0.001
DIC	13 (5.4%)	12 (15.0%)	7.651	0.006
MODS	44 (18.3%)	43 (53.8%)	38.018	<0.001
PCIS ≤80 points	57 (23.6%)	46 (57.5%)	31.311	<0.001
PRISM IV	3 (0,8.8)	10 (6,15)	−7.223	<0.001
PIM-3	−5.2(−6.7,−2.0)	−1.8(−2.3,−0.9)	−6.970	<0.001
Mechanical ventilation	59 (24.6%)	39 (48.8%)	16.493	<0.001
CRRT	6 (2.5%)	6 (7.5%)	2.886b	0.089
Death	10 (11.8%)	10(20.4%)	7.111	0.008

ALT, alanine aminotransferase; DIC, disseminated intravascular coagulation; MODS, multiple organ dysfunction syndrome; PCIS, pediatric critical illness score; PRISM IV, pediatric risk of mortality IV score; PIM−3, pediatric index of mortality version 3; CRRT, continuous renal replacement therapy; ^b^Continuity correction.

**Table 6:** Comparison of disease severity and prognosis of septic shock between the Two diagnostic criteria.

Clinical values	2005-criteria (*n* = 86)	2024-criteria (*n* = 49)	χ^2^/Z	*P*-value
Platelet count (×10^9^ /L)	167.0 (28.8,294.3)	40 (12.5,174.0)	−2.792	0.005
Albumin (g/L)	33.6 (29.4,38.0)	31.5 (27.2,34.5)	−2.327	0.02
ALT (U/L)	27.9 (17.1,55.1)	31.7 (16.9,70.6)	−0.666	>0.05
Creatinine (μmol/L)	27.2 (20.8,43.0)	36.9 (21.8,61.2)	−1.554	>0.05
Lactate ≥2 mmol/L	61 (70.9%)	36 (73.5%)	0.1	>0.05
Lactate ≥5 mmol/L	18 (20.9%)	17 (34.7%)	3.079	>0.05
DIC	12 (15.1%)	11 (22.4%)	1.594	>0.05
MODS	40 (46.5%)	39 (79.6%)	14.072	<0.001
PCIS ≤80 points	38 (44.2%)	28 (57.1%)	2.097	>0.05
PRISM IV	9 (4.8,15)	13 (8,17.5)	−2.949	0.003
PIM-3	−1.6(−2.2, −0.90)	−1.1(−2.0, −0.5)	−2.020	0.043
Mechanical ventilation	35 (40.7%)	24 (49.0%)	0.870	>0.05
CRRT	6 (7.0%)	6 (12.2%)	0.518b	>0.05
Death	10 (11.6%)	10(20.4%)	1.907	>0.05

ALT, alanine aminotransferase; DIC, disseminated intravascular coagulation; MODS, multiple organ dysfunction syndrome; PCIS, pediatric critical illness score; PRISM IV, pediatric risk of mortality IV score; PIM−3, pediatric index of mortality version 3; CRRT, continuous renal replacement therapy; ^b^Continuity correction.

This study has several limitations. The 2024 version of the diagnostic criteria was released in 2024, whereas our study commenced in 2023; therefore, all pediatric patients admitted to the intensive care unit were enrolled according to the 2005 criteria. Although the 2024 criteria are more stringent, and most children with sepsis or septic shock would have been included under this version, it is possible that a very small number of cases were missed, which may have introduced some bias into the statistical results. Future prospective studies enrolling patients separately according to the two versions of criteria are needed for more accurate comparison. In addition, subsequent work should involve conducting more high-quality studies and integrating approaches such as artificial intelligence to develop early warning models for the identification of pediatric septic shock. Such models would provide clinicians with objective predictive tools for the early recognition of septic shock in children.

## 5 Conclusion

The 2024 diagnostic criteria for sepsis are more stringent than those of the 2005 version, which helps avoid both overdiagnosis and overtreatment of sepsis while effectively identifying pediatric patients with a high risk of severe disease. However, in the diagnosis of septic shock, the 2024 criteria appear overly strict, making early identification and prompt intervention for pediatric septic shock more difficult. In clinical practice, pediatricians may use the 2024 criteria to screen for sepsis in children. Nevertheless, when recognizing septic shock, reliance solely on the PSS score is insufficient; close monitoring of heart rate, blood pressure trends, and tissue perfusion status—including mental status, urine output, skin signs, capillary refill time, and lactate levels—is essential to ensure timely intervention.

## Data Availability

The raw data supporting the conclusions of this article will be made available by the authors, without undue reservation.

## References

[1] Schlapbach LJ Watson RS Sorce LR Argent AC Menon K Hall MW International consensus criteria for pediatric sepsis and septic shock. JAMA. (2024) 331(8):665–74. 10.1001/jama.2024.0179 38245889 PMC10900966

[2] Goldstein B Giroir B Randolph A.; international consensus conference on pediatric sepsis. International pediatric sepsis consensus conference: definitions for sepsis and organ dysfunction in pediatrics. Pediatr Crit Care Med. (2005) 6(1):2–8. 10.1097/01.PCC.0000149131.72248.E6 15636651

[3] Singer M Deutschman CS Seymour CW Shankar-Hari M Annane D Bauer M The third international consensus definitions for sepsis and septic shock (sepsis-3). JAMA. (2016) 315(8):801–10. 10.1001/jama.2016.0287 26903338 PMC4968574

[4] Rudd KE Johnson SC Agesa KM Shackelford KA Tsoi D Kievlan DR Global, regional, and national sepsis incidence and mortality, 1990–2017: analysis for the global burden of disease study. Lancet. (2020) 395(10219):200–11. 10.1016/S0140-6736(19)32989-7 31954465 PMC6970225

[5] Liu G Zhang CM Li Y Sun JY Cheng YB Chen YP Respiratory virus infection and its influence on outcome in children with septic shock. Zhonghua Er Ke Za Zhi. (2024) 62(3):211–7. 10.3760/cma.j.cn112140-20231014-00286 38378281

[6] Wang S Yin F Zhang YY An K Xi YL Lu XL Epidemiology and clinical characteristics of pediatric sepsis in PICUs of China: a national cross-sectional study. MedComm (2020). (2023) 4(1):e211. 10.1002/mco2.211 36789098 PMC9921814

[7] Bone RC Balk RA Cerra FB Dellinger RP Fein AM Knaus WA American College of chest physicians/society of critical care medicine consensus conference: definitions for sepsis and organ failure and guidelines for the use of innovative therapies in sepsis. Crit Care Med. (1992) 20(6):864–74. 10.1097/00003246-199206000-00025 1597042

[8] Levy MM Fink MP Marshall JC Abraham E Angus D Cook D International sepsis definitions conference.2001 SCCM/ESICM/ACCP/ATS/SIS international sepsis definitions conference. Intensive Care Med. (2003) 29(4):530–8. 10.1097/01.CCM.0000050454.01978.3B 12664219

[9] Ren H Sun SJ Wang Y. Highlights and difficulties in the diagnosis and treatment of pediatric septic shock. Zhonghua Er Ke Za Zhi. (2025) 63(3):217–9. 10.3760/cma.j.cn112140-20241118-00839 39979094

[10] Subspecialty Group of Emergency Medicine, the Society of Pediatrics, Chinese Medical Association, Subspecialty Group of Pediatrics, the Society of Emergency Medicine, Chinese Medical Association, Editorial Board, Chinese Journal of Pediatrics. Expert consensus on the management of pediatric septic shock (2025). Zhonghua Er Ke Za Zhi. (2025) 63(3):220–9. 10.3760/cma.j.cn112140-20241106-00794 39979095

